# Timeliness of notification systems for infectious diseases: A systematic literature review

**DOI:** 10.1371/journal.pone.0198845

**Published:** 2018-06-14

**Authors:** Corien Swaan, Anouk van den Broek, Mirjam Kretzschmar, Jan Hendrik Richardus

**Affiliations:** 1 Centre for Infectious Disease Control, National Institute for Public Health and the Environment (RIVM), Bilthoven, the Netherlands; 2 University Medical Centre Utrecht, Utrecht, the Netherlands; 3 Department of Public Health, Erasmus MC, University Medical Center Rotterdam, Rotterdam, the Netherlands; The University of Hong Kong, CHINA

## Abstract

**Introduction:**

Timely notification of infectious diseases is crucial for prompt response by public health services. Adequate notification systems facilitate timely notification. A systematic literature review was performed to assess outcomes of studies on notification timeliness and to determine which aspects of notification systems are associated with timely notification.

**Methodology:**

Articles reviewing timeliness of notifications published between 2000 and 2017 were searched in Pubmed and Scopus. Using a standardized notification chain, timeliness of reporting system for each article was defined as either sufficient (≥ 80% notifications in time), partly sufficient (≥ 50–80%), or insufficient (< 50%) according to the article’s predefined timeframe, a standardized timeframe for all articles, and a disease specific timeframe. Electronic notification systems were compared with conventional methods (postal mail, fax, telephone, email) and mobile phone reporting.

**Results:**

48 articles were identified. In almost one third of the studies with a predefined timeframe (39), timeliness of notification systems was either sufficient or insufficient (11/39, 28% and 12/39, 31% resp.). Applying the standardized timeframe (45 studies) revealed similar outcomes (13/45, 29%, sufficient notification timeframe, vs 15/45, 33%, insufficient). The disease specific timeframe was not met by any study. Systems involving reporting by laboratories most often complied sufficiently with predefined or standardized timeframes. Outcomes were not related to electronic, conventional notification systems or mobile phone reporting. Electronic systems were faster in comparative studies (10/13); this hardly resulted in sufficient timeliness, neither according to predefined nor to standardized timeframes.

**Conclusion:**

A minority of notification systems meets either predefined, standardized or disease specific timeframes. Systems including laboratory reporting are associated with timely notification. Electronic systems reduce reporting delay, but implementation needs considerable effort to comply with notification timeframes. During outbreak threats, patient, doctors and laboratory testing delays need to be reduced to achieve timely detection and notification. Public health authorities should incorporate procedures for this in their preparedness plans.

## Introduction

Monitoring infectious diseases is essential for detecting outbreaks that demand public health response and control measures. Therefore, efficient and reliable surveillance and notification systems are vital for monitoring public health trends and early detection of disease outbreaks [1]. Timeliness is an important indicator for evaluation of surveillance systems, and defined as ‘reflecting the speed between steps in a public health surveillance system’ [2].

**Fig 1:**
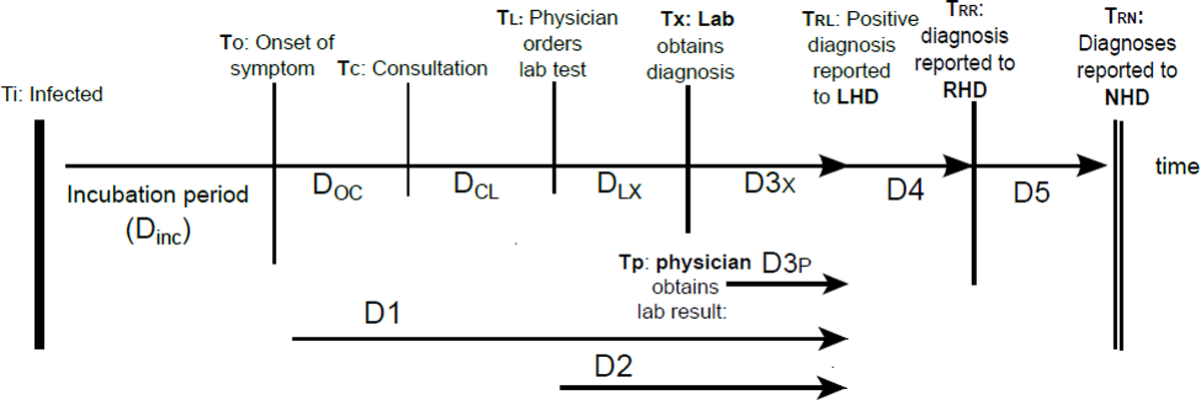
Notification timeline. D: delay; T: time point; D1: delay between onset of disease and notification at local health department (LHD); D2: delay between ordering a laboratory confirmation test and notification at LHD; D3X and D3P: delays between laboratory conformation test result and notification at the LHD by the laboratory and by the physician respectively; D4: delays between notification at LHD and reporting at regional health department (RHD); D5: delay between reporting at RHD and the national health department (NHD). Arrows: delays used in this article.

Public health response relies amongst others on notification of infectious diseases; a notifiable disease is a disease that is reportable either by law or by regulation [3]. Notification is the result of a chain of events from infection until report at the public health services, either local, regional or national [4]. Fig 1 illustrates the reporting timeline of infectious diseases. Delays in this chain are disease specific and the result of 1) patient delay, i.e. time elapsed from onset of disease until consultation of a physician (D_OC_), 2) doctors delay, time elapsed between consultation and ordering a laboratory confirmation test (D_CL_), and 3) laboratory delay, i.e. time elapsed until confirmation test result, depending on duration and frequency of testing (D_LX_). Lastly, there is a notification delay, from either laboratory or physician to the local health department (D3X and D3P, respectively), and reporting delay to regional and/or national health institutes (D4, D5 respectively). Most countries have installed legal obligations for physicians and diagnosing laboratories to notify certain infectious diseases to public health authorities according to a designated timeframe to ensure timely response, and in order to comply with international regulations [5, 6].

Notification systems traditionally involved conventional methods using postal mail, telephone, fax and/or electronic mail. Over the last two decades, electronic software systems for laboratory test recording and patient file records facilitated the development of electronic reporting systems, as electronic laboratory reporting (ELR), and automated ELR. [7] These electronic software systems have improved timeless of notification to public health services, both on local level as regional or national level [8–13]. Nowadays, inter-operable, interconnected, electronic real-time reporting systems have become the standard, and included as indicator for real time surveillance in the 2016 Joint External Evaluation (JEE) Tool of the WHO [14]. These systems however, are costly and evaluations of the surveillance systems reveal that also electronic reporting systems do not always meet the designated (‘predefined’) notification timeframes [15, 16].

There is a lack of international reviews on which factors related to notification systems influence timeliness of reporting of infectious diseases. In this study, a systematic review of peer-reviewed literature was performed to assess timeliness of notification systems. In order to determine factors associated with timely notification, we compared timeliness of notification systems in three ways: firstly using the ‘predefined timeframe’, i.e. the timeliness criteria designated by the study itself, secondly using a ‘standardized timeframe’, i.e. identical timeliness criteria for all studies designated for this review, and thirdly using ‘disease specific timeframes’, i.e. timeliness criteria differentiated between specific diseases.

## Methodology

### Search strategy and selection criteria

**Fig 2:**
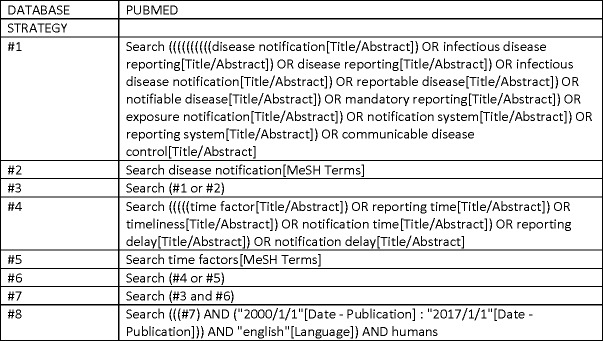
Full electronic search strategy for pubmed.

A systematic review was conducted using the PRISMA framework (Preferred Reporting Items for Systematic Reviews and Meta-analyses). Articles reviewing timeliness of infectious disease notification systems, published during January 1^st^ 2000—January 1^st^ 2017 were included. Earlier articles were excluded to avoid information on outdated notification systems. A detailed search strategy in biomedical and public health literature was conducted in two electronic databases, Pubmed and Scopus, using a combination of free-text search terms and medical subject headings. The search included terms related to infectious disease reporting (‘disease notification’, ‘notification system’, ‘infectious disease reporting’, ‘exposure notification’, ‘communicable disease control’) and reporting timeliness (‘reporting time’, ‘notification time’, ‘reporting delay’, ‘time factor’). The date last searched was January 30^th^ 2017. The full electronic search strategy for Pubmed is depicted in Fig 2.

The identified articles from each literature search were reviewed on title and abstract. Studies published in English, during the period 2000–2017, and concerning human infectious diseases (in general or disease specific) were included. Excluded were studies that only described notification completeness or only described the timeliness between symptom onset and diagnosis, or focused on notification compliance of healthcare professionals, or described timeliness of reporting from national to international health organizations. In addition, studies describing a surveillance algorithm, and studies which did not provide information about the designated criteria for timeliness of notification, i.e. the ‘predefined timeframe’, were excluded. Studies without predefined timeframe, but comparing timeliness of different notification systems were included. Systematic reviews were excluded, however their conclusions are reflected upon in the discussion.

One researcher (AB) reviewed all titles and abstracts. In case of doubt about inclusions or exclusion, another researcher (CS) was consulted and through discussion a decision was taken.

Subsequently, references were imported into the bibliographic database Endnote library, where duplicates were identified and removed. The remaining articles were reviewed in full text to determine their inclusion for data extraction. Reference lists of the included articles and reviews were searched for additional literature.

### Data extraction

Information extracted included the country or region of the study setting, year of publication, infectious disease(s), general or disease specific reporting system, study design (comparison study where two or more reporting methodologies were compared, or evaluation study when one system was evaluated), level of reporting and methodology of reporting, legislation (mandatory or voluntary reporting), reporting delay studied, predefined timeframe for reporting and the outcomes of the reporting delay(s). The following categorizations were made:

Level(s) of reporting:
  - level 1 (L1): physician and/or laboratory to local public health department (LHD);
  - level 2 (L2): LHD to regional health department (RHD);
  - level 3 (L3): RHD to national health authority (NHA).
Method of reporting:
  - conventional reporting (postal mail, fax, telephone or e- mail);
  - electronic reporting (including web-based reporting systems, as electronic laboratory reporting (ELR), electronic automated laboratory reporting (EALR).
  - mobile phone reporting (using shore message services with mobile telephones)
Reporting delay (see Fig 1):
  - D1: delay between onset of disease and notification at local health department (LHD);
  - D2: delay between ordering a laboratory confirmation test and notification at LHD;
  - D3X and D3P: delays between laboratory conformation test result and notification at the LHD by the laboratory and by the physician respectively; in case the study did not differentiate between reporting either through laboratory or physician, the delay was defined as D3P/X;
  - D4: delays between notification at LHD and reporting at regional health department (RHD);
  - D5: delay between reporting at RHD and the national health department (NHD).

For each selected study, one researcher extracted the relevant data.

### Timeframes and classification of study outcomes

WHO defines reporting timeliness as the proportion of all expected reports in a reporting system received by a given date [17]. We evaluated the timeliness results of the notification system of each study according the following timeframes:

1. The predefined timeframe: the timeliness criteria designated by the study itself. These are defined through legislation, local rules or by the authors of that specific study. In case authors used a different timeframe for analyzing than the mandatory timeframe, we followed the authors’ decision.
2. The standardized timeframe: in order to analyze equally the relation between the timeliness outcomes and notification systems of the different studies, we defined as standardized timeframe: D1 ≤ 14 days, D2 ≤ 7 days, D3 (including D3P, D3X and D3P/X) ≤ 1 day, D4 + D5 (D4/5) ≤ 5 days and D1-5: ≤21 days. We chose rather strict delays for D3 and D4/5 as these can be reasonably achieved by a well-functioning notification system. Less strict delays were chosen for D1 and D2 as they are related to patient and doctor’s delay, availability and duration of laboratory test, which differ per infectious disease.
3. The disease specific timeframe: as timely intervention to prevent or control an outbreak is disease specific, we defined disease specific median reporting delays between onset of disease and notification at the local health department (D1). These were calculated for timely control measures to reduce the proportion of infection caused by secondary cases to outbreak control levels (‘optimal’ and ‘suboptimal’ conditions) as determined by Bonacic et al [4]: for hepatitis A median ≤ 8 or ≤ 17 days, hepatitis B ≤ 1 or ≤ 42 days, measles ≤ 2 or ≤ 5 days, mumps ≤ 3 or ≤ 8 days, pertussis ≤ 4.5 days (only criteria for suboptimal conditions available) and for shigellosis ≤ 1 and ≤ 3 days.

Timeliness outcomes of the reporting system in each study were classified as follows: score ≥ 80% of notifications in time: ‘sufficient’, in line with the WHO JEE Tool which recommends timeliness of reporting at least 80% of all reporting units [14]. Scores between ≥50% and < 80% notifications in time were classified as ‘partly sufficient’, and scores < 50% of notifications in time as ‘insufficient’ as we consider the system functioning improperly when more than half of all notifications are not within the timeframe.

Several included studies presented timeliness outcomes of different delays in the notification system, outcomes of different (groups) of diseases or outcomes of different notification systems, within the same study. In case these outcomes involved different scores a mixed score was given: either ‘sufficient/partly sufficient’, or ‘sufficient/ insufficient’, or ‘partly sufficient/insufficient’. When different outcomes in time were reported in a follow-up study, we chose the most recent outcome for scoring as this usually was the best and final result of a notification system. In intervention studies, we chose the outcome of the most successful intervention for scoring. In case a study presented outcomes of multiple reporters in different geographic areas, the outcome ≥ 80% of the reporters was used for scoring.

Subsequently, factors associated with timely notification systems were assessed. In addition, in studies comparing different notification systems, these outcomes were assessed separately. In intervention studies, timeliness of the different reporting systems was compared to identify factors related to timeliness.

## Results

**Fig 3:**
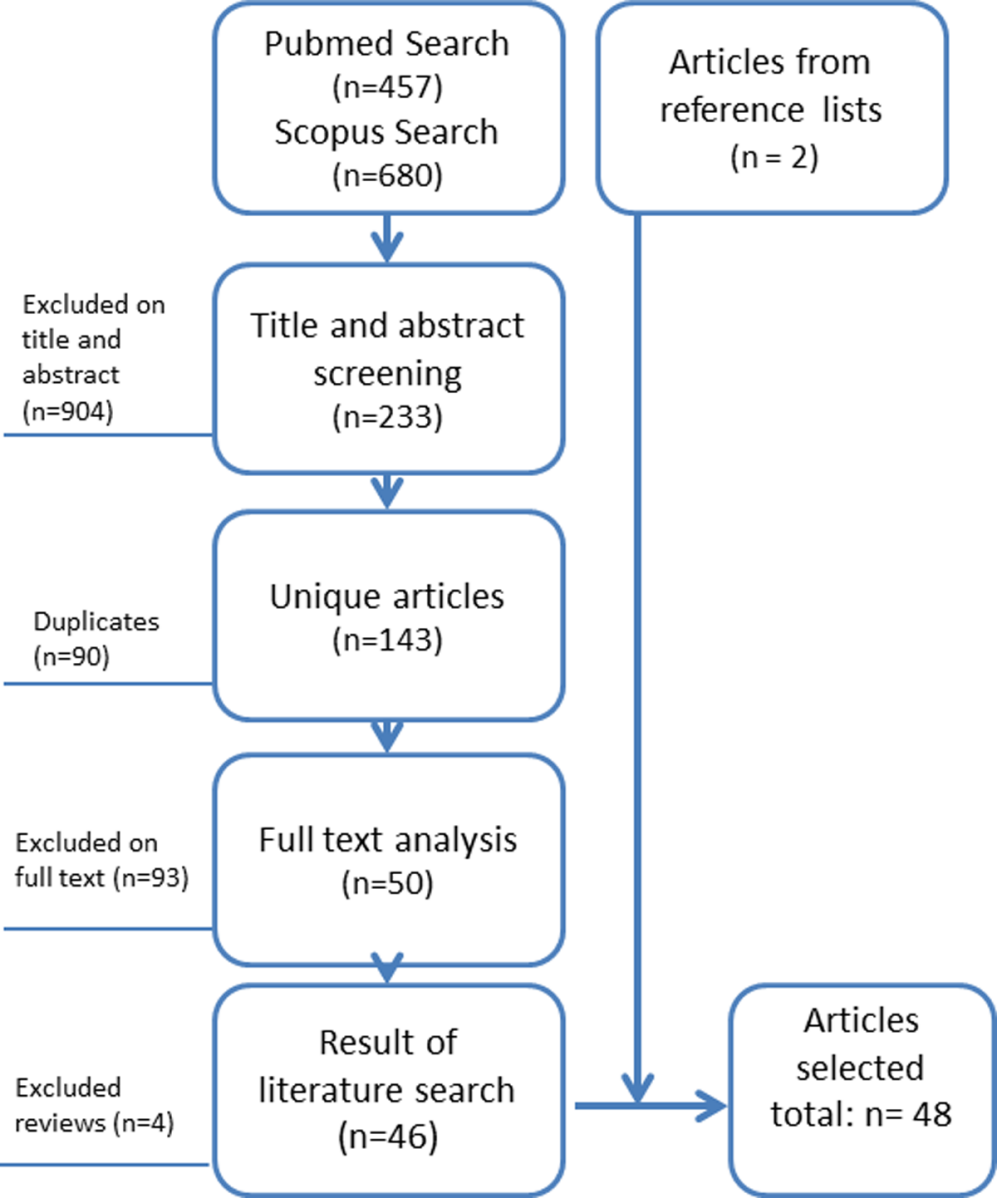
Flow diagram search process.

An overview of the search process is depicted in the flowchart in Fig 3. In total 48 articles were included in the review [3, 9–11, 13, 15, 16, 18–58]. An overview of study characteristics and results is shown in Table 1. The articles involve notification systems in 17 countries, mainly Northern America (United States 20 studies), Europe (14 studies) and East Asia (6). The majority of the studies (27 studies) analyze the timeliness of notification of one specific infectious disease, either in a disease specific notification system (13) or a generic notification system (14). Groups of infectious diseases were analyzed in 21 studies, one study analyzed timeliness of reporting of several syndromes.

**Table 1:** Overview of study characteristics and results.

			Notification system					
Study number, author, year country	Disease(s), Disease specific system +/ -	Study*	Level of reporting**	Method of reporting***	Reporting Delay described #	Predefined timeframe	Timely according predefined timeframe? S-P-I ##	Timely according standardized timeframe? S-P-I ##
1. Altmann et al. 2011, Germany ^18^	STEC (+)	I	Level 1,2,3 Mandatory	L1: D3P **C** (fax, telephone, post). D3X? L2-L3?	D1, D3P/X, D4 + D5	D3P: < 24hr D4-5: <16 days	D3P: P, D4-5: S	D3P: P D4-5: S
2. Begier et al. 2005, US, Connecticut ^19^	Anthrax (+)	E	Level 1 Mandatory	**C**: Telephone	D3X	D3X: Immediately	D3X: P	D3X: P
3. Carrieri et al. 2000, Italy ^20^	25 diseases	E	Level 2,3 Mandatory	**E** (SIMI system: Electronic Computerized System)	D4-5	D4-5: One month per level	D4-5: I	D4-5: I
4. Choe et al. 2014, Republic of Korea ^21^	Measles (+)	I	Level 1 and 2–3 Mandatory	?	D1, D3P/ X, D4-D5	Mandatory: D3P: ≤7 days, D4-5: ≤1 day. Study: D3P/X: ≤ 1 day. D4-5: ≤ 1 day.	D3 P/X: I D4-5: S	D3 P/X: I D4-5: S
5. Curtis et al. 2001, US (6 states) ^22^	TB (-)	E	Level 1 and 2	C	D3P/D3X, D4	D3P/X: ≤ 2 days, D4: ≤ 1 day, (CDC) D3-4: ≤ 3 working days.	D3-4: I	D3-4: I
6. Day et al. 2007, UK ^23^	Gastro- enteritis (-)	E	Level 1 Mandatory	Formal:? informal: telephone /C	D3P	Clinical notification directly; before lab confirmation.	D3P: I	D3P: I
7. Freeman et al. 2013, UK ^24^	Several ID	I	Level 1 Mandatory	E	D2	D2: Report <21 days after earliest specimen date. Lab is timely if >90% of reports is timely.	D2: I	D2:I
8. Garcell et al. 2014, Qatar ^25^	Several ID	I	Level 1 Mandatory	**C**: Telephone or Fax	D1, D3P	D3P: notification delay: Group1: 24h Group2: 72h	D3P: G1: I G2: S	D3P: G1: I G2: S
9. Ghosh et al. 2008, US, Colorado ^26^	Influenza (+)	E	Level 1 Mandatory and Voluntary	**E** ‘CERDS’ and **C**: Fax (passive), telephone (active)	D3P/ D3X	Not available	Comparative study	
10. Goto et al. 2016, Brazil ^27^	Dengue (-)	E	Level 1, Mandatory	E ‘SINAN’, online computerized notification system	D1	D1 Study: ≤ 7 days	D1: S	D1: S
11. Grills et al. 2010, Australia ^28^	Campylobacter (-)	E	Level 1 Mandatory	**C**: Post (mainly lab), fax (mainly MD), telephone (little).	D3P, D3X	D3P/X Notification ≤ 5 days	D3P: S D3X: P	3DP: P D3X: I
12. Haller et al. 2014, Germany ^29^	Healthcare associated outbreaks (+)	E	Level 2,3 Mandatory	C: fax, e-mail	D4-5	D4:Within 3 working days D5: ≤ 1 week	D4: S, D5: S	D4: I, D5: I
13. Heisey-Grove et al. 2011, US, Massachusetts ^30^	Hepatitis C (+)	I	Level 1 Voluntary	C (paper) -> E: (electronic reporting forms)	D3P	Not available	Comparative study	
14. Huaman et al. 2009, Peru ^31^	Several ID	E	Level 1 Voluntary	**C**:Telephone, radio, **E**: elec-tronic surveillance system	D3P	Immediately after detection/ mandatory twice a week.	D3P clinics: S, ships: P	D3P: P
15. Jajosky et al. 2004 US ^3^	Several ID	E	Level 1,2, 3 Mandatory	C and E	D1, D3P, D3X, D4, D5	Within 1 or 2 incubation periods	D1-D5 ≤1 or 2 IP^a^: I, D3-5: ≤1 or 2 IP: I	D1-D5 ≤1 or 2 IP: I, D3-5: ≤1 or 2 IP: I
16. Jansson et al. 2004, Sweden ^9^	4 ID	E	Level 1, Mandatory	E: ‘SmiNet’ (computerized reporting system) vs C: paper-based	D2, D3P, D3X	D3P:signed notification within 24 h of diagnosis	D3P: I	D3P: I
17. Johnson et al. 2014, US, Oklahoma ^32^	Several ID	E	Level 1 Mandatory	**E** (automated ELR) compared with **C** (fax/mail/phone)	D3P/X	Within 1 business day	D3PX: Conv: P, E:S	D3P/X: S
18. Kite-Powell et al. 2008, US, Florida ^33^	4 ID	E	Level 1, Mandatory	C (fax, mail, phone) compared with ELR theoretical	D1, D3X	D1% reported within 1 or 2 incubation periods	D1 ≤ 2 IP^a^ (both C and E theoretical): P	D1 ≤ 2 IP (both C and E theoretical): P
19. Lo et al. 2011, Taiwan ^34^	TB (+)	E	Level 1 Mandatory	L1:? (C?) L2 Web-based (E)	D3P	Mandatory: ≤ 7 days of suspicion/ confirmation. Study: ≤ 7 days of start treatment.	D3P: S	D3P: I
20. Mc Kerr et al. 2015, Taiwan, ^35^	Dengue (-)	E	Level 1 Mandatory	**E:** Web-based: NDSS	D3P, D3X	Within 24 hr	D3P/X: S	D3P/X: S
21. Mlynarski et al. *2*009, US, Connecticut ^36^	Anthrax (+)	E	Level 1 Mandatory	**C**: telephone, (besides fax, postal mail)	D3X	<12 or < 24 hr: when ≤ 32hr of culture growth gram + rods.	D3X ≤ 12 hr: P, ≤ 24hr: S	D3X: ≤ 12 hr: S, ≤ 24hr: S
22. Moore et al. 2008, US, New York City, ^37^	Hepatitis A (-)	E	Level 1 Mandatory	**C/ E**: ELR (1->35% in study period)	D3P, D3X	Timely enough to provide PEP to contacts (≤ 10 days of diagnosis of index)	D3P/X: S	D3P/X: I
23. Murray et al. 2013, US, California ^38^	Gonorrhoeae (-)	E	Level 1 and 2 Mandatory	C	D3P, D3X	D3P within 7 days. D3X within 1 business day. D4: weekly reporting	D3P: I	D3P: I
24. Nazzal et al. 2011, Qatar ^39^	Measles (-)	E	Level 1,2,3 Mandatory	C: Notification forms	D1 –D5	WHO recommendation: 80% < 2 days.	D1-5: I	D1-5: S
25. Nguyen et al. 2007, US, New York City, ^40^	Several ID	E	Level 1 Mandatory	E (ECLRS Electr clin lab report system) compared with paper reports.	D2	Not available	n.a.^b^	D2: P
26. Overhage et al. 2008 US, Indiana, ^11^	Several ID	E	Level 1 Mandatory	E: Automated ELR compared with C: paperbased	D3X	Not available	n.a.	n.a.
27. Panackal et al. 2002, US, Pennsylvania ^13^	10 ID	E	Level 1 Mandatory	E: Automated ELR compared with C: paperbased	D3X	Not available	n.a.	D3X: P
28. Paranthaman et al. 2009, UK ^41^	Meningo-coccosis (-)	E	Level 1 Mandatory	C/E: Paper and electronic forms	D3P	Immediately reporting to LHD. Indirect: same day of admission	D3P: I	D3P: P
29. Pascopella et al. 2004, US, California ^42^	TB (-)	E	Level 1 Mandatory	unknown	D3X	Within 1 working day.	D3X: S	D3X: S
30. Quan et al. 2014, South Africa ^43^	Malaria (+)	I	Level 2, Mandatory	C: paper forms. SMS/text messages	D3P, D4+5	D3P < 24 hrs. D4+5 <72 hrs 2 days between reporting and follow up.	D3P, D4+5:P	D3P: P, D4+5: S
31. Rajeev et al. 2011, US, Utah ^44^	Several ID	E	Level 1 Mandatory	E: Electronic case reporting HL7 vs paper-based (comparison)	D2	Immediately or within 3 working days depending on disease.	D2: P	D2: S
32. Ratnayake et al, 2013, Canada ^45^	Meningococcosis (-)	E	Level 1 Mandatory	C: Telephone and fax	D2 (P&L)	Mandatory ‘prompt’, in study predefined timeframe: 7d	D2: S	D2: S
33. Reijn et al. 2008, Netherlands ^46^	6 ID	E	Level 1 and L2-3 Mandatory	**C** L1: Fax, phone, paper card, L 2-3/D4-5: **E** webbased.	D1, D3P, D4-5	D3P: ≤ 1 day, or ≤3 days when weekend interferes. D4-5: over-night. Study timeframe: 1–2 IP	D1, D3P: P	D1, D3P: P
34. Richard et al. 2008, Switzerland ^47^	Measles (+)	E	Level 1 Manda-tory (MNS) vs Voluntary (SSSN)	C: e-mail	D1	L1/D2: MNS: clinical compatible cases: < 1 week	D1: I	D1: P
35. Riera-Montes et al. 2011, Sweden ^48^	Chlamydia (-)	E	Level 1 Mandatory	E: Electronic surveillance system: case based reporting and lab. (SmiNet 2)	D3P, D3X	24 hours	D3P/X: I	D3P/X: I
36. Rosewell et al. 2013, Papua New Guinea^58^	Several syndromes	E	Level 1 Voluntary	MR: MOPBASSS vs C (paper)	D3P	Once weekly	D3P: P	D3P: I
37. Samoff et al. 2013, US, North Carolina ^49^	Several ID	I	Level 2 and 3, Mandatory	E: ELR vs C: fax/mail	D4 –D5	Not available	n.a.	D4-5: I
38. Severi et al. 2014, UK, London SE ^50^	Salmonella (-)	E	Level 1 Mandatory	E prereporting and C prereporting (fax, tel, postal/ email)	D1, D2	D3: Within 7 days. (but not used)	n.a.	D1: P
39. Silin et al. 2010, US, NYC ^51^	TB (+)	I	Level 1 Mandatory	C: e-mail (fax)	D3P, D3X	Within 24h	D3P/X: P	D3P/X: P
40.Stachel et al. 2014, US, NYC ^52^	Several ID	E	Level 1, Mandatory	E (ELR) and C (fax, mail, phone)	D2	Not available	n.a.	D2: P
41. Sun et al. 2016, China ^53^	Malaria (+)	E	Level 1, Mandatory	E: NIDRIS, internet based reporting	D3P, D3X	Within 1 day	D3P/X: S	D3P/X: S
42. Tosti et al. 2015, Italy ^54^	Hepatitis (+)	E	Level 1, 3 Man-datory + Volun-tary (SEIEVA)	E:Web-based reporting	D3P, D3-5, D2	D3 within 48h of diagnosis (mandatory).	D3P: I	D3P: I
43. Troppy et al. 2014, US, Massachusetts ^55^	Several ID	E	Level 1 Mandatory	Automated ELR, EHealthR	D4	Not available	n.a.	D4: I
44. Vogt et al. 2006, US, Colorado ^15^	Several ID	E	Level 1, L2 Mandatory	E (CERDS in LHD) or C: Fax, e-mail, phone	D2 (speci-men col-lection rep)	24h or 7 day, depending on disease	D2: ≤ 1 day: I, ≤ 7 days: S	D2: S
45. Ward *et al*. 2005 Netherlands ^10^	Several ID	E	Level 1,2,3 Mandatory (1)	L1: C fax, phone, e-mail. L2-3: C -> E: ELR	D1, D4-D5	D4-5: as soon as possible.	D4-5: P	D4-5: S
46. Xiaqiang et al. 2011, China, Yunnan ^56^	Hepatitis A (-)	E	Level 1,2, 3 Mandatory	Online, real-time web-based reporting.	D3, D4-D5	D3 <1 day, D4: < 1 day	D3P: S	D3P: S
47. Yoo et al. 2009, Republic of Korea ^16^	6 ID	E	Level 1,2, 3 Mandatory	E: Electronic reporting system	D1 D3P,4,5	D3,4,5: either < 1 day or < 7 days (depending on ID)	D3P, 4, 5 ≤ 1 day: P, ≤ 7 days: S	D3P, D4: P, D5: S
48. Zucs et al. 2005 Germany ^57^	Several ID	E	Level 1, 2 Mandatory	L2-L3: E: ERS, L1: C:	D3X	D3X within 24h	D3X: S	D3X: S

*: E = Evaluation, I = Intervention

**: L1: physician and/or laboratory to local health department (LHD); L2: LHD to regional health department (RHD); L3: RHD to national health authority (NHA).

***: Method of reporting: C: Conventional (postal mail, fax, telephone, e-mail), E: Electronic (webbased applications, f.e. (automated) electronic laboratory reporting)). MR: mobile phone reporting

#: See Fig 1. D3 P/X: notification delay either by physician or by laboratory to local health department

##: S: sufficient; P: partly sufficient; I: insufficient

a: IP: incubation periods, b: n.a.: not applicable

There were 40 evaluation studies, of which 19 studies included a comparison of notification methods, and 8 intervention studies. Mandatory reporting is most common in notification systems (42 studies), next to voluntary reporting (3 studies) or a combination of both (3 studies). Most studies described reporting at local level (L1, 31 studies), followed by a combination of local and regional/national level (L1-L2 and/or L1-L2-L3, 13 studies). Four studies report on regional and/or national level (L2 and/or L2-L3). The studies analyzed conventional reporting methods (13 studies), electronic reporting (10), a combination of both (20), or mobile phone reporting (2). Three studies did not provide information on the reporting methodology, and were excluded in the analyses of timeliness related to reporting systems. Reporting delay on local level, including the delay between physician or laboratory to the local health department after laboratory confirmation (D3) was studied (43 studies) most often. Only 5 studies focused on delay towards regional or national level. An overview of delays reported in 48 articles is illustrated in the supporting information S1 table.

### Timeliness

Out of 48 studies, 39 provided a predefined timeframe. Nine studies without predefined timeframe provided a comparison between outcomes of different notification systems. In total 35 out of 39 studies with a predefined timeframe referred to a quantitative, and 4 studies to a qualitative timeframe (‘immediate’, ‘as soon as possible’), see Table 1. Quantitative timeframes involved numbers of days/weeks/months, incubation periods per infectious disease (3 studies), or period for effective post exposure prophylaxes for contacts (1 study). The most common predefined timeframe for D3 P/X was reporting ≤ 1 day (12 studies), or ≤ 32–48 hour (3 studies). Predefined timeframes for notification on local level varied considerably between ≤ 1 day and ≤ 3 weeks, on regional/national level between ≤ 1 day and ≤ 2 months.

**Fig 4:**
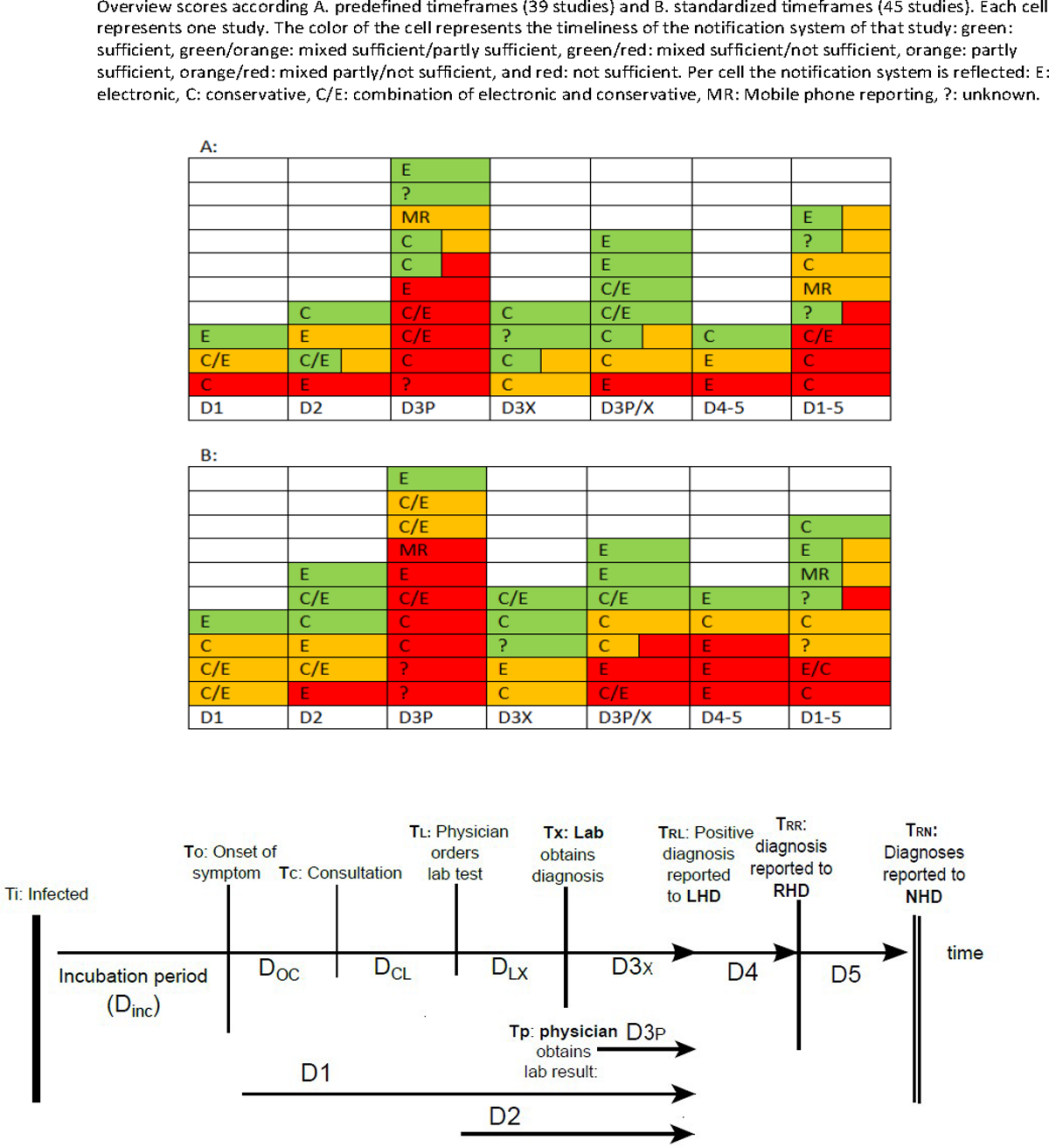
Overview scores according predefined and standardized timeframes.

In 11 of the 39 studies (28%), notification delays met the predefined timeframe, in 12 (31%) not, and in the other 16 studies the outcomes were partly sufficient (8, 21%) or a mixed score (8, 21%). In Fig 4 these outcomes are visualized according to the delay described in a study, including information on the notification system. Notification systems involving the laboratory (D3X or D3X/P) showed the best results: 3 out of 4 (D3X) and 5 out of 7 (D3P/X) studies had sufficient or mixed sufficient/partly sufficient timeliness according their predefined timeframe. Notification systems only involving physicians (D3P) showed least favourable results: in 5 out of 10 studies the timeliness was insufficient according their predefined timeframes.

In 34 of these 39 studies, information on the notification system(s) was provided and involved in 13 studies conventional methods, in 10 studies electronic methods, in 9 studies a combination, and in 2 studies mobile phone reporting. As shown in Fig 4, there appeared to be no relation between notification system and score. Of the eleven studies were the notification system scored sufficient, four studies used a longer predefined timeframe for delays for D3 and D4-5 [29, 34, 37, 45], and four studies with a strict predefined timeframe (D3 or D4 <1 day) used an electronic notification system. Three of the latter studies were conducted in East Asian countries. Both Chinese and the Taiwanese studies revealed sufficient notification [34, 35, 53, 56]. In the 12 studies with an insufficient notification system, three out of four studies with a strict timeframe (D3 < 1 day) used either conventional or electronic reporting [9, 23, 41]. Notification systems in three out of these 12 studies with insufficient scores described D1, i.e. 25%, [3, 39, 47], while in total only 5/38 studies included D1, i.e. 13%. Eight out of twelve studies were from Europe (Italy, UK, Sweden and Switzerland) [9, 20, 23, 24, 41, 47, 48, 54].

For analyzing notification systems according to standardized timeframes, 45 studies were included (Fig 4). In 13 studies (29%), the system was scored sufficient, in 15 studies (33%) not, and in the other 17 studies the outcomes were partly sufficient (13, 29%) or a mixed score (4, 9%). 8 studies scored better related to the standardized timeframe, 8 studies scored worse. Sufficient notification systems frequently involved D1, D2 and D3X (8/13). Insufficient notification systems involved frequently physicians (D3P) (7/15) and public health authorities D4-5 (5/15). In parallel with the outcomes of the predefined timeframes, no clear relation between scoring result and notification system could be observed. Although the distribution of outcomes in both timeframes was comparable (24/38), some studies did score differently according to predefined or standardized timeframes: 3/12 studies scoring an insufficient notification system for the predefined timeframe improved in scoring for the standardized, while 3/11 studies changed from sufficient to partly or not sufficient.

With regard to the disease specific timeframe, 8 studies provided information regarding delay D1 for one or more specific diseases. In none of them the notification system was timely enough for optimal outbreak control. Suboptimal outbreak control was shown for notification systems for hepatitis A [10, 33, 46], hepatitis B [25] and measles [11]. However the system was insufficient for outbreak control in most studies: hepatitis A [25], measles [21, 25, 46, 47], pertussis [10] and shigelloses [15, 46, 52].

### Comparison and intervention studies

In 13 studies timeliness of electronic systems was compared with conventional systems. In the majority (10/13) electronic reporting was faster than conventional reporting, improving timeliness with days (range 0–11) [9–11, 13, 33, 40, 44, 49], up to months [20, 30]. However, none of these studies fulfilled the predefined timeframe, and only 2 the standardized timeframe [10, 44]. In 3 studies, conventional reporting method was as fast as, or faster than electronic systems [26, 32, 50].

Six studies analyzed a variety of interventions in the notification systems: increased frequency (daily reporting[18]), sentinel lab surveillance [21], legal adjustments [24], training [25] and better facilities (fax), SMS text messages [43] and systematic monitoring delayed reports (conventional reporting) [51]. In all studies timeliness improved (range several days), however, none of the interventions resulted in sufficient timeliness for predefined or standardized timeframes.

## Discussion

To our knowledge, this is the first systematic review assessing timeliness of notification systems. Thirty-nine out of 48 identified studies from 17 different countries provided quantitative data including a predefined timeframe. Timeliness of almost one third of the systems was sufficient, one third insufficient and the others partly sufficient, both for the predefined as the standardized timeframes. Reporting delay by laboratories, either combined with by physicians, was timelier than other delays in the notification chain in both timeframes. Outcomes were not related to notification systems. Although electronic systems were faster in comparative studies (10/13), this hardly resulted in sufficient scorings for theirs systems, neither according predefined nor standardized timeframes. The disease specific timeframe for optimal outbreak control was not met by any study.

Notification systems for infectious diseases are country, or even state/province, specific and therefore difficult to compare [3]. However, the studies in this review demonstrate that many components of the notification chain (Fig 1) are generic, including indicator based reporting on local, regional/national level, reporting by treating physician and/or diagnosing laboratory at local level, and mostly involving legally mandatory notification according to quantitative timeframes (hours, days, weeks). Remarkably, 29 out of the 48 studies involved the delays from physician and/or laboratory to the local health authorities (D3P, D3X or D3X/P). The predefined timeframes, either mandatory or chosen by the authors, for this delay where also quite comparable; for example 13 studies used a timeframe of ≤ 1 day. Nevertheless, differences in predefined timeframes do exist; therefore we introduced in this review a standardized timeframe per delay in order to compare notification timeliness between studies. We choose for standardized timeframes delays that were achievable. Eight studies had no timeframe. Although the overall outcome between using the predefined timeframe and the standardized timeframe was comparable, as is shown in Fig 4, the outcomes of over one third of the studies (14/38) changed by applying the standardized timeframe. In our opinion, the outcomes of applying a standardized timeframes are most representative in the appraisal of timeliness of a notification system.

It is remarkable that studies provide little background explanation about the designated timeframes, except when incubation periods are used, which are considered to be related to communicability and therefore critical when considering control measures [3, 46], or when timeframes related to measures such as post exposure prophylaxis are used [37]. The purpose of notification systems in general is an early warning system to identify outbreaks, to enable public health authorities to take corrective action through effective preventative and/or control measures, and to monitor the effect of implemented measures [48]. Timely notification for this purpose is disease specific, as we have demonstrated earlier [4]. Using available data on notification delays in the Netherlands for six person-to-person communicable diseases, at the time of reporting to local public health services, over 80% of secondary cases already were infected by the index. Therefore timely notification will mainly prevent tertiary, and further, cases. In none of the 8 studies in this review that provided relevant information regarding D1 medians for these 6 diseases, the notification system was timely for effective outbreak control. This might be one of the reasons why infectious diseases such as measles are difficult to control and still are endemic in many industrialized countries.

Another aspect is that only certain parts of the notification chain can be influenced through the notification system: mainly the reporting of a confirmed infectious disease from laboratory and/or physician to the local health department (D3), and from here to regional and/or national level (D4-D5). Timeliness outcomes for these delays were less sufficient than for D1-D2 in the standardized system. This review shows that many notification systems therefore can be improved to minimize delays D3 and D4-5. However, patient delay to consult a physician is not related to a notification system, neither the doctor’s delay in recognizing a disease. As patient, doctor’s and laboratory delays (D1,D2) take longer time than notification delays D3, D4, D5, (S 1 Table) optimizing notification systems will only partly optimize the timeliness of the entire notifications chain. Reduction of patient, doctor’s and laboratory delays, through increased awareness and enhanced availability of laboratory tests, is essential to substantially improve timeliness of the notification chain. This is certainly indicated in situations of increased threats. In such situations, also temporary conventional notification methods as telephone calls to the local health departments have an added value. Therefore decisions on investments in notifications systems should take into consideration the reduction in timeliness in D3, D4-D5 compared to potential reduction of D1-D2 and D3 (telephone) in case of specific health threats.

Although this was not the primary aim of the study, we identified the following facilitators and barriers related to timeliness outcomes of notification systems:

1. Concerning reporters (physicians, laboratories): facilitating factors: motivation, communication (between public health services and reporters), awareness raising, acceptance and simplicity of procedures and clinical guidelines, knowledge, training, phone call reminders, regular feedback [3, 9, 16, 25, 31, 32, 36, 38, 45, 54]. Barriers were lack of knowledge, lack of communication, uncertainty towards notification procedures [39, 45].
2. Available resources: availability of staff, technical facilities (fe fax) and rapid laboratory transport [25, 27, 42]. Barriers were different laboratory software among laboratories and using out-of-state laboratory facilities [38, 53, 57].
3. Notification procedures: unification of reporting times, legal adjustments of notification time, f.e. to frequency of reporting, a centralized data base, periodically evaluation of the system and analyses of delayed reports [15, 16, 18, 23, 24, 42, 47]. Barriers were administrative procedures and high volume of cases [39].
4. Others: higher number of notifiable cases during an epidemic was reported as barrier [28], but considered facilitating factor in others as extra supportive staff was made available. [27, 47] Public education is a facilitator to reduce patient delay [16].

Although we cannot come to conclusions to which extent these barriers and facilitators influence the timeliness of notification systems, it is obvious that addressing these aspects contribute to optimized functionality of the system.

Over the last two decades, several studies demonstrated the value of electronic reporting systems reducing notification delays [7]. However, over the last years, implementation of ER also revealed challenges. Gluskin *et al*. summarize in their systematic literature review that ELR, comparable with results of our study, reduces reporting time on average with 8.5 days (range 4–17 days) [59]. Besides increased volumes of incomplete notifications, coding of infectious diseases can be a challenge for laboratories when adjusting diagnostic tests, and for public health authorities whose computer systems have to keep up with de ELR codes. Also considerable information technology infrastructure, expertise and workforce need to be available for a good operating system, requiring substantial financial investments. The next step forward would be notifications through Electronic Medical Records (EMR), also requiring technical and financial investments, but addressing the physician reporting delay (D3P), which had the lowest scores in timeliness in our review. This system also can combine clinical systems and several laboratory tests resulting in notifications complying with case definitions which will reduce the workload for both public health services and physicians considerably. [60] Another interesting development in rural, resource poor settings is the use of mobile phone reporting. The studies of Quan et al and Rosewell et al showed that mobile phone reporting using SMS, shortened reporting time compared with conventional paper-based reporting and follow up from 37 to 7 days (medians) and from 84 to 2.4 days (averages) in South Africa and Papua New Guinea respectively [43, 58]. This methodology is simple, user friendly, reliable, and technically feasible in rural areas. It might be interesting to consider the use of mobile phone texting in addition to existing sophisticated notification systems in situations of newly emerging diseases or enhanced surveillance in high income countries as well.

### Limitations

Studies used different parameters to calculate timeliness of their notification systems. In case the median, percentiles or means were used, we had to classify the score according to the percentage of notifications within timeframes. In case of doubt, or when the score was close to the cutoff of 50% or 80%, a second author was consulted to come to a decision. Also the opinion of the authors of the study reflected in the paper was used to come to a score.

Some studies used the delay between specimen collection at the laboratory and notification at the local health departments. These delays were included as D2 as well, even though the test result was not yet available, in order to limit the number of different delays used in this study. It is noteworthy that 8 studies, while presenting the delays of their notification system, did not include a predefined timeframe, either mandatory or chosen by the authors of the study. Also in several studies there was a difference between the mandatory timeframe and the timeframe chosen by the authors, without explanation. A realistic mandatory timeframe should be developed. It might be good to add a standardized timeframe, at least for D3 and D4-5, mostly affected by the notification system, in the Joint External Evaluation tool by the WHO.

The cut-offs in the scoring and delays in the standardized timeframe have been chosen on the above described grounds, but still are based on the opinions of the authors of this study. We consider 80% of timely notifications demonstrating a sufficient system, and 80% is in line with the WHO standard for an indicator based surveillance system [14], however an early warning system with 1/5 notifications not timely can cause considerable effect on effective control measures. When applying a 90% score as sufficient, the studies with sufficient timely notification systems almost halved from 11 (29%) to 6 (16%). Therefore we comply with the WHO standard.

In several articles, different notification systems were mentioned, both conventional and electronic, without clarifying which notifying organization used which system. In that case we classified the system as combined conventional/electronic (C/E). Therefore, with the limited number of selected articles, this review might not have shown an existing difference between conventional and electronic systems.

Lastly, we did not include completeness of notifications (percentage notified diseases) or completeness of information provided in the notification. We are aware that certain aspects of notification systems facilitate completeness, for example ELR towards notification completeness, and physician reporting to completeness of information provided. We refer readers to the many articles and reviews written on this subject.

## Conclusion

This systematic review shows that a minority of notification systems meet either predefined, standardized or disease specific timeframes. Systems which include laboratory reporting, either combined with reporting by physicians, are more often associated with timely notification. Electronic reporting systems are not associated with sufficient timeliness of notifications, while they need a considerable investment. And, even when fully implemented, they will only reduce a part of the notification chain, excluding D1-D2. Therefore, during outbreak threats, patient, doctors and laboratory testing delays need to be reduced to achieve timely detection and notification. Conventional reporting methods, like phone calls, and mobile phone texting, still can play an important role, besides alerting potential patients, physicians, and provision of appropriate laboratory test. Public health authorities should be aware of these aspects and incorporate contingency systems for enhanced notification in their preparedness plans.

## Supporting information

S1 Table Delays of notifications system per study and timeliness of notification system according author’s predefined timeframe. (TIFF)

S2 Table Prisma 2009 checklist. (DOC)
