# Supplementary figures and images for: Timeliness of notification systems for infectious diseases: A systematic literature review
Source: PLoS One. 2018 Jun 14;13(6):e0198845. doi: 10.1371/journal.pone.0198845 (PMC6002046; doi:10.1371/journal.pone.0198845)

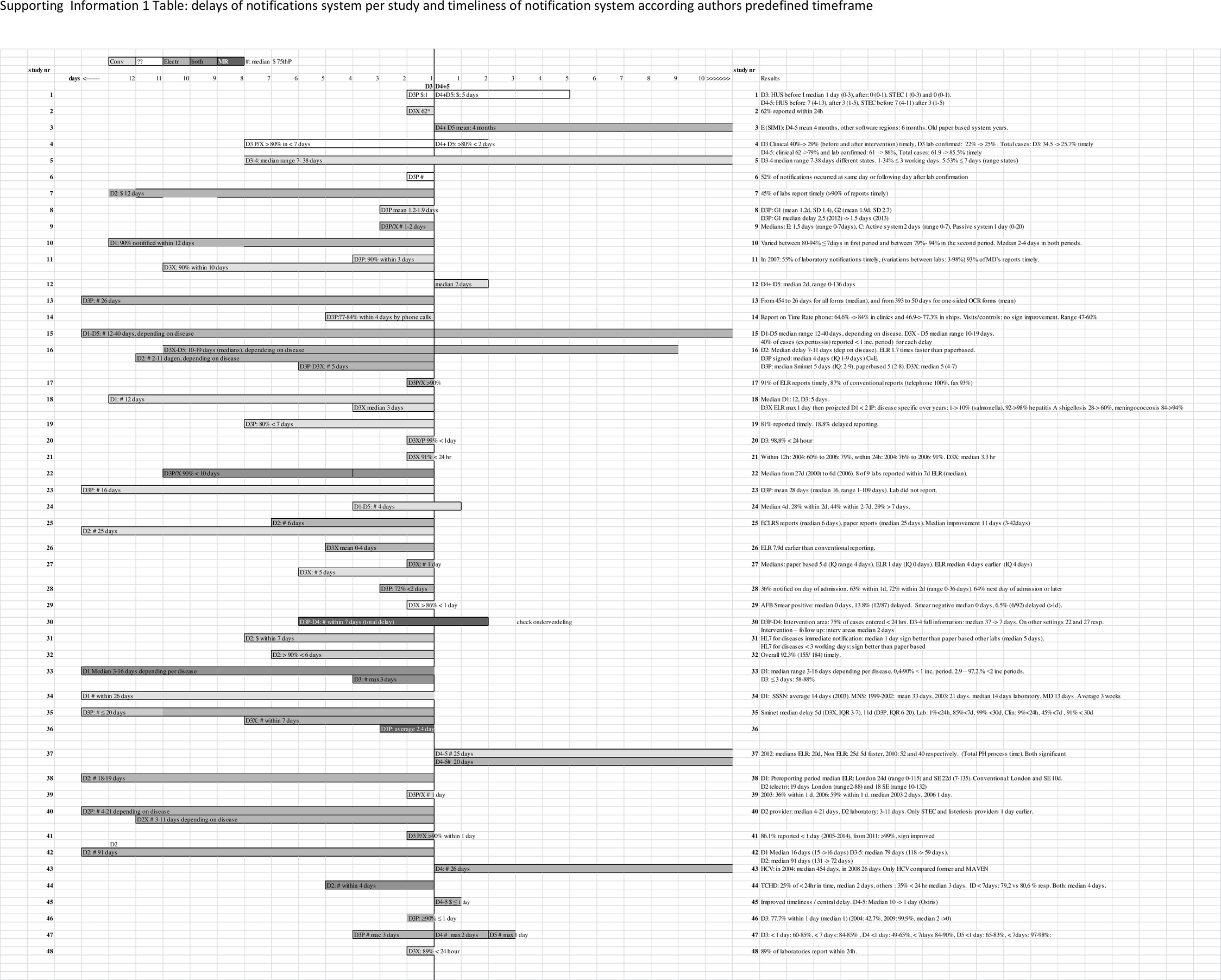

Supplement: S1 Table — (TIFF) [file pone.0198845.s001.tiff]
